# Intestinal Drug Absorption Enhancement by *Aloe vera* Gel and Whole Leaf Extract: In Vitro Investigations into the Mechanisms of Action

**DOI:** 10.3390/pharmaceutics11010036

**Published:** 2019-01-18

**Authors:** Anja Haasbroek, Clarissa Willers, Matthew Glyn, Lissinda du Plessis, Josias Hamman

**Affiliations:** 1 Centre of Excellence for Pharmaceutical Sciences (Pharmacen™), Potchefstroom Campus, North West University, Potchefstroom 2520, South Africa; 22692592@nwu.ac.za or anjahaasbroek11@gmail.com (A.H.); 20672322@nwu.ac.za (C.W.); Lissinda.duPlessis@nwu.ac.za (L.d.P.); 2 Preclinical Drug Development Platform (PCDDP), Potchefstroom Campus, North West University, Potchefstroom 2520, South Africa; mglynmglyn@live.co.uk

**Keywords:** *Aloe vera*, gel, whole leaf, absorption enhancement, Caco-2, confocal laser scanning microscopy, F-actin, FITC-dextran, tight junctions, transepithelial electrical resistance

## Abstract

The co-administration of absorption enhancing agents with macromolecular drugs (e.g., protein and peptide drugs) has been identified as a means to improve the oral bioavailability of these drugs. Absorption-enhancing agents of natural origins have received a great deal of attention due to their sustainable production, in support of green chemistry. In previous studies, certain parts of the *Aloe vera* leaf (e.g., gel and whole leaf extract) have shown a potential to enhance drug permeation across the intestinal epithelial barrier. The mechanism of the drug-absorption-enhancement action and the capacity for absorption-enhancement of the *A. vera* gel and whole leaf, were investigated in this study. A clear decrease in transepithelial electrical resistance (TEER) of Caco-2 cell monolayers exposed to *A. vera* gel and wholeleaf extract, in various concentrations, indicated the opening of tight junctions between the epithelial cells. The transport of Fluorescein isothiocyanate (FITC)-dextran, with a molecular weight of 4 kDa (FD-4), could be enhanced across the Caco-2 cell monolayers, by the *A. vera* gel and whole-leaf extract, but not the FITC-dextran with larger molecular weights (i.e., 10, 20, and 40 kDa), which indicated a limited drug absorption enhancement capacity, in terms of the molecular size. Accumulation of FD-4 between the Caco-2 cells (and not within the cells), after treatment with the *A. vera* gel and whole-leaf extract was shown with a confocal laser scanning microscopy (CLSM) imaging, indicating that the paracellular transport of FD-4 occurred after the interaction of the *A. vera* gel and whole-leaf extract, with the epithelial cell monolayers. Furthermore, changes in the F-actin distribution in the cytoskeleton of the Caco-2 cell monolayers was observed by means of a fluorescence staining, which confirmed tight junction modulation as the mechanism of action for the absorption enhancement effect of the *A. vera* gel and whole-leaf extract.

## 1. Introduction

The oral route of drug administration is associated with relatively high patient compliance and is more affordable, when compared to the injection therapies [1]. Reasons for a high patient compliance with the oral route of administration, include self-treatment, ease of use, and its non-invasive nature [2]. On the other hand, oral drug administration is challenged by the low bioavailability of certain drugs, such as macromolecular drugs [1]. The general low-membrane permeability and oral bioavailability of large compounds (molecular weight > 500 Da) can be ascribed to their unfavorable physico-chemical properties [3], as well as the harsh gastrointestinal environment where enzymatic and chemical activity cause extensive degradation, especially, of protein and peptide drugs [4].

For an orally administered drug to have its desired pharmacological effect, the drug must reach the systemic circulation via absorption, through the intestinal epithelial layer [5], which can occur via the paracellular or transcellular pathways [6,7]. The paracellular pathway is the transport of drug molecules between epithelial cells and occurs by means of size-limited passive diffusion, through the tight junctions and intercellular spaces. Hydrophilic macromolecules, such as peptide and protein drugs are mainly transported via the paracellular route, since they cannot penetrate cell membranes [7,8], however, their paracellular movement is severely restricted by the tight junctions between the adjacent epithelial cells [9]. A promising approach to improve the oral absorption of these hydrophilic macromolecules is the co-administration of absorption enhancers [7].

Tight junctions (zonula occludens) are one of three intercellular complexes, with adherence junctions (zonula adherens) and desmosomes (macula adherens), which link epithelial cells together. Tight junctions can be described as dynamic multi-protein complex structures consisting of various transmembrane proteins, with the main proteins being occludin, tricellulin, and the claudin family. These proteins are connected to the cell actin cytoskeleton, via the scaffolding protein zonula occludens-1 (ZO-1). Thus, a change in the actin distribution can be linked to a modulation of one or more tight junction proteins [10,11]. The dynamic nature of the tight junction ensures that it can be modulated by different stimuli, resulting in an increased paracellular absorption, in a reversible and potentially safe manner [12]. Tight junction modulation can be experimentally confirmed by transepithelial electrical resistance (TEER) measurements, as well as the permeability of paracellular markers. In addition, microscopic examination after staining of cell components and intercellular accumulation of fluorescent probes, can be used to indicate the opening of tight junctions as a mechanism of paracellular drug-absorption enhancement [13,14].

Oral drug-absorption enhancement of protein and peptide drugs can be defined as the process of improving the movement of the drug molecules across the intestinal epithelium, which can be accomplished by the incorporation of functional excipients, in dosage forms. This improved membrane permeation should be accomplished without damaging the cells or causing toxic effects [15,16]. Absorption enhancers are chemical adjuvants that are co-administered with peptides and proteins, to increase their bioavailability, by reversibly removing or disrupting the intestinal barrier, with minimal tissue damage [17]. The mechanisms through which this can be achieved include decreasing mucus viscosity, changing membrane fluidity, disrupting the structural integrity of the intestinal wall or modulating the tight junctions [17,18].

Many compounds have already been investigated for their potential drug-absorption enhancing abilities, including various chemicals of natural origin that are derived from plants (capsaicin, piperine, quercetin, and *Aloe vera*) and from animals (chitosan and zonula occludens toxin). Different mechanisms of absorption enhancement have been suggested for these chemical absorption-enhancing agents, such as regulation of gastrointestinal function, enzyme inhibition, P-gp efflux inhibition, mucoadhesion, and tight junction modulation [12,19,20,21,22,23,24].

*Aloe vera* is a succulent perennial xerophyte that displays the water-storage mechanisms in leaves, such as the formation of a viscous mucilage to survive in arid regions with little or irregular rainfall [25,26]. The innermost part of the leaf is made up of clear, moist, soft, and slippery tissue that consists of thin-walled parenchyma cells [27]. As a result, the thick fleshy leaves contain amongst other compounds, storage carbohydrates, such as acetylated mannan (acemannan or aloverose) and cell wall carbohydrates, such as cellulose and hemicellulose [28].

*Aloe vera* has long been used in traditional medicine, where the latex has been used for its laxative effects and the gel was mainly used for the treatment of wounds and skin ailments, such as psoriasis and genital herpes [29]. Other uses that have also been ascribed to *A. vera* components include anti-bacterial, anti-cancer, anti-diabetic, anti-fungal, anti-obesity, anti-viral effects, and gastric protection against ulcers [25,29,30].

A study was conducted to evaluate the effect of *A. vera* liquid preparations on the absorption of vitamin C and E, in human subjects, and it was found that *A. vera* markedly improved the oral bioavailability of both these vitamins [31]. It was shown in an in vitro study that the *A. vera* gel and whole-leaf extract had the ability to markedly enhance the transport of insulin across the Caco-2 cell monolayers [32]. Thereafter, several in vitro studies were conducted on the effect of the *A. vera* gel and whole-leaf extract on macromolecular and other hydrophilic compounds, across intestinal epithelial cell monolayers, excised intestinal tissues [32,33,34,35], excised skin [36,37], and across excised buccal mucosa [38]. The P-gp-modulating effects of the *A. vera* juice was investigated by Djuv and Nilsen [39], but they found that the *A. vera* juice did not inhibit the P-gp mediated transport of digoxin, in a statistically significant way in any of the concentrations that were tested.

The aim of this study was to identify the mechanism of action by which the *A. vera* gel and the whole leaf extract, enhance the gastrointestinal absorption of macromolecules, as well as to establish the capacity of these materials in terms of the size of the molecules, which can be moved across the intestinal epithelium. This was done by determining the transport of the FITC-dextran with different molecular weights across the Caco-2 cell monolayers, after treatment with the *A. vera* gel and whole-leaf extract, by TEER studies, by visualization of the accumulation of the FITC-dextran (4 kDa) between the Caco-2 cells, on the monolayers grown on membrane inserts, and by fluorescence staining and visualization of the F-actin structure of the Caco-2 cells, after incubation with the *A. vera* gel and the whole-leaf extract.

## 2. Materials and Methods

### 2.1. Materials

Dehydrated *Aloe vera* gel powder 200X (Dalton Max 700^®^ gel) and whole leaf, decolourised, spray-dried *Aloe vera* powder 100X (whole-leaf extract) were kindly donated by Improve USA. Inc. (De Soto, TX, USA). Chitosan (ChitoClear^®^ with a degree of deacetylation of 96% and viscosity of 8 cP for a 1% solution) was purchased from Primex (Siglufjordur, Iceland). Fluorescein isothiocyanate (FITC)-dextran with molecular weight (MW) of 4 kDa (FD-4), 10 kDa (FD-10), 20 kDa (FD-20), and 40 kDa (FD-40), as well as Lucifer Yellow, were purchased from Sigma-Aldrich/Merck (Darmstadt, Germany). CytoPainter^®^ Phalloidin iFluor 488 and Fluoroshield^®^ mounting medium, with propidium iodide, were purchased from Abcam (Cambridge, MA, USA).

### 2.2. Chemical Characterisation of the A. vera Gel and the Whole-Leaf Extract

Quantitative proton nuclear magnetic resonance (^1^H-NMR) analysis was used to chemically characterise the *A. vera* gel and the whole-leaf extract, by determining the content of certain marker molecules, including aloverose, glucose, malic acid, and iso-citric acid (whole-leaf marker), as previously described [40].

### 2.3. Chemical Characterisation of N-Trimethyl Chitosan Chloride

*N*-trimethyl chitosan chloride (TMC) was synthesised from chitosan (ChitoClear^®^, degree of deacetylation of 96% and viscosity of 8 cP, for a 1% solution), as previously described [41], and characterized by the means of ^1^H-NMR spectroscopy, using an Avance III 600 Hz NMR spectrometer (Bruker BioSpin Corporation, Rheinstetlen, Germany). A sample of the TMC (100 mg) was dissolved in 2 mL D_2_O and analyzed in the NMR spectrometer, at 80 °C, with a suppression of the water peak. The degree of quaternization of the TMC was calculated from the ^1^H-NMR spectra, by using Equation (1), as previously described [42]:

(1) $$\mathrm{DQ}\;\left(\%\right)\;=\;\left[\left(\frac{\int^{\;}\mathrm{TM}}{\int^{\;}H}\right)\;\times \;\frac{1}{9}\right]\;\times \;100$$

where DQ (%) is the percentage of the degree of quaternization, ∫TM is the integral of the trimethyl amino group (quaternary amino) peak at 3.7–4.0 ppm, on the ^1^H-NMR spectra, and ∫H is the integral of the ^1^H peaks from 4.7–6.2 ppm, on the ^1^H-NMR spectra.

### 2.4. Caco-2 Cell Culturing

The Caco-2 cells were procured from the European Collection of Authenticated Cell Cultures (ECACC). Caco-2 cells were cultured in a growth medium that consisted of high-glucose Dulbecco’s Modified Eagles Medium (DMEM), supplemented with 10% *v*/*v* fetal bovine serum (FBS), 1% *v*/*v* non-essential amino-acid solution (NEAA), 1% *v*/*v* penicillin/streptomycin (10,000 U/mL penicillin and 10,000 U/mL streptomycin), 1% *v*/*v* amphotericin B (250 μg/mL), and 2 mM l-glutamine. The cells were incubated at 37 °C and exposed to 95% humidified air and 5% CO_2_. The Caco-2 cells were used between passages 51–56.

### 2.5. Cell Monolayer Integrity

The integrity of the cell monolayers was confirmed by measuring the TEER, as well as determining the permeation of the exclusion marker, Lucifer yellow [43,44].

Prior to each permeation experiment, the TEER of the Caco-2 cell monolayers was measured to confirm the formation of a confluent cell monolayer on the insert membrane. The minimum TEER values, as indicated in Table 1, were required and considered as indicative of the presence of intact cell monolayers, in the different Transwell^®^ plates (Corning Costar^®^, Corning, NY, USA) [45,46,47].

Lucifer yellow was used as an exclusion transport marker molecule, to confirm the formation of confluent Caco-2 cell monolayers on the inserts of each Transwell^®^ plate. The growth medium was aspirated from the basolateral chambers of the 6-well Transwell^®^ plate and replaced with the appropriate volume of pre-heated serum-free DMEM, buffered with *N*-(2-hydroxyethyl) piperazine-*N*-(2-ethanesulfonic acid) (HEPES) (pH = 7.4), and incubated for 30 min, at 37 °C. After 30 min, the Transwell^®^ plates were removed from the incubator, the growth medium was aspirated from the apical chambers and replaced with an appropriate volume of a pre-heated Lucifer yellow solution (i.e., 50 µg/mL in serum-free DMEM) [44]. The Transwell^®^ plates were incubated with the Lucifer yellow solution and samples (200 µL) were withdrawn from the basolateral chamber, every 20 min, for 120 min, and replaced with equal volumes of pre-heated serum-free DMEM buffered with HEPES. The Lucifer yellow concentration in the samples was quantified by means of fluorescence spectroscopy, at excitation, and at emission wavelengths of 485 nm and 535 nm, respectively [48]. The percentage transport of Lucifer yellow across the Caco-2 cell monolayer should be less than 2% for the two-hour transport period, to indicate intact cell monolayers [48]. Furthermore, apparent permeability coefficient (P_app_) values of the Lucifer yellow ≤ 0.2 × 10^−6^ cm/s [43] or 0.66–0.75 × 10^−6^ cm/s [44], were considered indicative of the formation of intact Caco-2 cell monolayers.

### 2.6. In Vitro Transepithelial Electrical Resistance (TEER) Study

Caco-2 cells were cultured in Transwell^®^ 24-well plates (Corning Costar^®^) on insert membranes, with a surface area of 0.33 cm^2^ and pore size of 0.4 μm, to form confluent monolayers. The positive control consisted of 0.5% *w*/*v* TMC (a known tight junction modulator), while the test solutions consisted of *A. vera* gel and whole-leaf extract, each in four different concentrations ranging from 0.1 % *w*/*v* to 1.5% *w*/*v*. Serum-free DMEM alone was used as the negative control.

The TEER measurements of the Caco-2 cell monolayers on insert membranes in 24-well Transwell^®^ plates commenced one hour prior to addition of the test solutions, to obtain the TEER values, at a baseline level. DMEM buffered with HEPES (pH = 7.4) (1 mL) was added to the basolateral chamber and incubated for 30 min, prior to the addition of the test solutions (200 µL) to the apical chamber on top of the cell monolayers, on the filter membranes. The TEER (T_0_) was measured directly, after application of the test solutions to the apical chamber. TEER measurements were then taken at 20 min intervals up to 120 min, after addition of test solutions. TEER was measured with a Millicell ERS meter (Millipore, Billerica, MA, USA) that was connected to a set of chopstick electrodes.

### 2.7. In Vitro Permeation Studies

Caco-2 cells were cultured in Transwell^®^ 6-well plates (Corning Costar^®^) on insert membranes, with a surface area of 4.67 cm^2^ and a pore size of 0.4 µm, to form confluent monolayers. For the in vitro permeation study, four different FITC-dextran (i.e., FD-4, FD-10, FD-20 and FD-40) solutions were prepared, each in a concentration of 125 μg/mL, in serum-free DMEM. Four different concentrations of each of the *A. vera* gel and whole leaf extract (ranging from 0.1 % *w*/*v* to 1.5% *w*/*v*) were added to each of the FITC-dextran solutions, to prepare the experimental solutions. Control groups consisted of each FITC-dextran in serum-free DMEM, without *A. vera* gel and the whole-leaf extract.

The in vitro permeation of each of the FITC-dextran molecules, in the absence and presence of the different *A. vera* gel and the whole-leaf extract solutions, was determined in the apical to basolateral (AP-BL, absorptive) direction, across the Caco-2 cell monolayers. First, the growth medium was aspirated from the basolateral chamber and replaced with 2.5 mL pre-heated serum-free DMEM buffered with HEPES (pH = 7.4) and placed back in the incubator (37 °C), to equilibrate for 30 min. After 30 min, the Transwell^®^ plates were removed from the incubator and the growth medium from the apical chamber was aspirated and replaced with 2.5 mL of each of the experimental solutions pre-heated to 37 °C. Samples (200 µL) were extracted from the basolateral chamber at 20 min intervals for a total period of 120 min and replaced with 200 µL pre-heated DMEM buffered with HEPES. The quantification of the FITC-dextran concentrations, in the samples, was done by means of fluorescence spectroscopy, at excitation and at emission wavelengths of 494 and 518 nm, respectively.

The percentage transport was calculated from the concentration of each FITC-dextran (i.e., FD-4, FD-10, FD-20, and FD-40) measured in the samples withdrawn from the basolateral chamber, at each time interval. The percentage transport was calculated with the following equation:

(2) $$\%\mathrm{Transport}\;=\;\frac{\mathrm{Drug}\;\mathrm{concentration}\;\mathrm{at}\;\mathrm{specific}\;\mathrm{time}\;\mathrm{interval}}{\mathrm{Initial}\;\mathrm{FITC}-\mathrm{dextran}\;\mathrm{dose}}\times 100$$

The apparent permeability coefficient (P_app_) values were calculated from the percentage transport data across the Caco-2 cell monolayers, for each of the FITC-dextran molecules. P_app_ is defined as the permeability rate that is normalized by the surface area, across which the permeation occurs, as well as the concentration, assuming the starting concentration in the acceptor (basolateral) chamber is zero [49]. The P_app_ was calculated by using the following equation [50,51]:

(3) Papp = dcdt1(A·60·C0),

where P_app_ is the apparent permeability coefficient (cm·s^−1^), (dc/dt) represents the permeability rate (concentration/min, represented by the slope of the transport curve), A is the permeation surface area (cm^2^), and C_0_ is the starting concentration of the permeant.

From the P_app_ values, the permeation-enhancement ratio (R) values were calculated by the following equation [32]:

(4) $$R\;=\;\frac{P_{\mathrm{app}}\;\mathrm{experiment}}{P_{\mathrm{app}}\;\mathrm{control}}$$

where R is the permeation-enhancement ratio, P_app_ experiment is the apparent permeability coefficient for the test solution, and P_app_ control is the apparent permeability coefficient for the control group.

### 2.8. Caco-2 Cell Monolayers for the Confocal Laser Scanning Microscopy (CLSM) Study

For the CLSM visualisation studies (for both the transport pathway and the F-actin filament studies), the Caco-2 cells were cultured in Snapwell^®^ 6-well plates (Corning Costar^®^), with removable filter-rings, which had a surface area of 1.12 cm^2^ and a pore size of 0.4 µm, to form confluent cell monolayers. Stock solutions of the FITC-dextran 4 kDa (FD-4, 1 mg/mL), the *A. vera* gel, the *A. vera* whole-leaf extract (2.0% *w*/*v*), and TMC (1.0% *w*/*v*), were each prepared, separately, in a serum-free DMEM. These stock solutions were used to prepare the experimental solutions, which consisted of combinations of the FITC-dextran and each of the permeation enhancers solutions (i.e., *A. vera* gel, *A. vera* whole leaf, and TMC) in a 1:1 ratio that were applied to the Caco-2 cell monolayers. The final concentrations of the test solutions were, therefore, 1.0% *w*/*v A. vera* gel, 1.0% *w*/*v A. vera* whole-leaf extract and 0.5% *w*/*v* TMC, while the final concentration of FITC-dextran (FD-4) in the mixture, applied to the cell monolayers, was 0.5 mg/mL. The negative control group consisted of serum-free DMEM, without any of the chemical permeation enhancers.

For the fluorescence staining, a 10× CytoPainter^®^ Phalloidin iFluor 488 solution was prepared by diluting 5 µL of a 1000× phalloidin conjugate in dimethyl sulfoxide (DMSO) stock solution with 500 µL PBS, containing 1.1% *v*/*v* foetal bovine serum (FBS). The 0.1% *v*/*v* Triton X100 solution was prepared by diluting 3 µL of the 100× Triton X-100 solution to 300 µL with phosphate buffer saline (PBS).

#### 2.8.1. Fluorescence Staining

##### Visualisation of the Transport Pathway

After 21 days of culturing, in the Snapwell^®^ 6-well plates, and confirmation of the cell monolayer formation, the cell monolayers were incubated with the above-mentioned experimental solutions for 2 h at 37°C, 5% CO_2_, and 95% air (i.e., the same conditions as for the in vitro permeation study).

After the 2 h incubation period, the cells were fixed with 4% formaldehyde for 10 min [52,53] and gently rinsed, once, with ice-cold PBS [54]. After fixation, the cell monolayers were prepared on the microscope slides, as described below in Section 2.8.2, and confocal images were taken with a Nikon Eclipse TE-3000 inverted microscope (Nikon Instruments, Melville, NY, USA), equipped with 60× and 100× ApoPlanar oil immersion objectives and a DSRi1 Nikon digital camera, for real-time imaging. The microscope was linked to a Nikon D-Eclipse C1 confocal system. The images were taken at room temperature, under light exclusion. All experiments were done in triplicates.

##### Visualization of the F-Actin Filaments in the Cytoskeleton

Staining of the F-actin in the cytoskeleton of the Caco-2 cells was used to identify if opening of tight junctions was the mechanism of action of the *A. vera* gel and the whole-leaf extract, in terms of drug-absorption enhancement [55,56]. The cell monolayers in the Snapwell^®^ 6-well plates were incubated with the experimental permeation enhancer solutions (*A. vera* gel and whole-leaf extract without FD-4) for 2 h, at 37 °C, 5% CO_2_, and 95% air. The cell monolayers were then fixed with 4% formaldehyde, for 10 min, and gently rinsed, once, with ice-cold PBS. Fixation was followed by permeabilization (to increase the accessibility of the F-actin to the CytoPainter^®^ Phalloidin iFluor probe) with 0.1% Triton X-100 for, 3 min, after which the cell monolayers were gently rinsed with PBS. Thereafter, F-actin staining was done with 10X CytoPainter^®^ Phalloidin iFluor 488 for 60 min and gently rinsed for 5 min, with PBS. The cell monolayers were prepared on the microscope slides, as described below in Section 2.8.2, and images were taken with CLSM, as described above. All experiments were done in triplicates.

#### 2.8.2. Preparation of the Microscope Slides for the Confocal Laser Scanning Microscopy (CLSM)

The filter-ring was removed from the Snapwell^®^ insert and placed onto a glass plate, to add support before the filter membrane was cut loose with a scalpel. The filter membrane was cut into smaller sections and a section with a size of, approximately, 1.12 cm × 0.3 cm was transferred to a microscope slide. Three to four drops of the Fluoroshield^®^ mounting medium, containing propidium iodide [57], were added and spread-out, evenly. Care was taken not to touch the cell monolayer on the filter membrane. The propidium iodide, contained in the mounting media was used to visualize the cell nuclei. Finally, the excess Fluoroshield^®^ mounting medium was removed by gently touching the slide with a piece of paper towel, and then a coverslip was added.

#### 2.8.3. Imaging with Confocal Laser Scanning Microscopy

The CLSM was equipped with an Argon Ion laser (emission wavelength of 488 nm or 515 nm), a Helium Neon polarised laser (emission wavelength of 543 nm), and a blue Diode laser (emission wavelength of 409 nm). The excitation and emission wavelengths used for the imaging of the FITC-dextran, Phalloidin iFluor, and the propidium iodide are shown in Table 2.

### 2.9. Data Analysis

Data analyses on the in vitro permeation results were performed with STATISTICA Version 12 (Statsoft, Tulsa, OK, USA, 2013). All data sets were subjected to the Brown-Forsythe test to establish the normality and homogeneity of the data distribution. Normally distributed data were analyzed by analysis of variance (ANOVA) with Dunnet’s post-hoc tests (two-sided). For data sets that were not normally distributed, non-parametric Kruskal-Wallis testing was applied. Statistically significant differences were accepted when *p* < 0.05.

## 3. Results and Discussion

### 3.1. Characterisation of the A. vera Gel and the Whole-Leaf Extract

The quantitative ^1^H-NMR analysis indicated that the *A. vera* gel contained 15.2% aloverose; 9.8% glucose; 2.0% citric acid, and 20.7% malic acid, while the *A. vera* whole-leaf extract contained 4.9% aloverose; 8.6% glucose, 8.9% citric acid, 24.7% malic acid, and 14.6% iso-citric acid, or whole-leaf marker [33].

### 3.2. Characterization of the N-trimethyl Chitosan (TMC)

**Figure 1:**
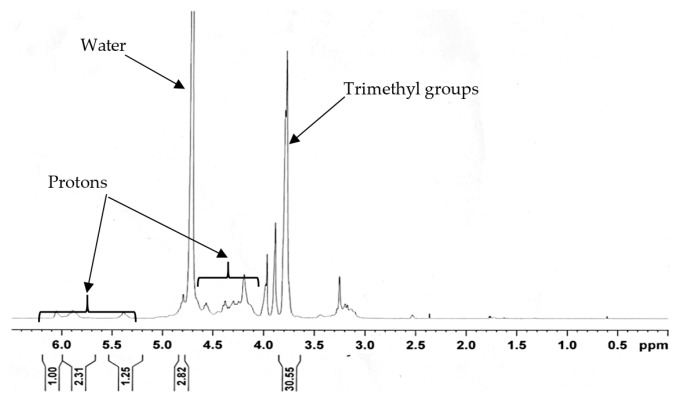
Proton nuclear magnetic resonance (^1^H-NMR) spectrum for the *N*-trimethyl chitosan chloride (TMC).

The degree of quaternization (DQ) of the TMC was calculated to be 45.995%, from the ^1^H-NMR spectrum of the TMC (Figure 1), using Equation (1).

### 3.3. Cell Monolayer Integrity Using Lucifer Yellow

The cumulative percentage transport of Lucifer yellow, across the Caco-2 cell monolayers was below 2%, over a period of 120 min, which indicated an acceptable integrity of the Caco-2 cell monolayers, as suggested by Wahlang et al. [48]. The apparent permeability coefficient (P_app_) value for the Lucifer yellow was calculated to be 0.346 × 10^−6^ cm/s, from the transport curve, which was also within the range of the suggested P_app_ values for the Lucifer yellow, when transported across the Caco-2 cell monolayers, with an acceptable integrity, namely 0.2–0.75 × 10^−6^ cm/s [43,44].

### 3.4. In Vitro Transepithelial Electrical Resistance (TEER) Study

**Figure 2:**
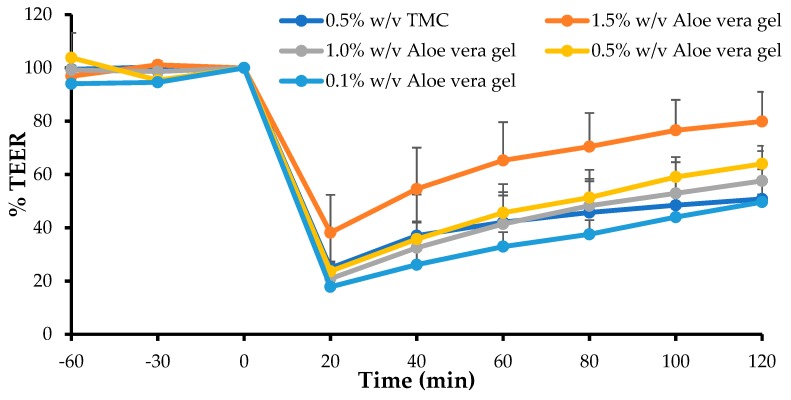
Percentage transepithelial electrical resistance (TEER) of the Caco-2 cell monolayers, after application of the *A. vera* gel, at different concentrations, and *N*-trimethyl chitosan chloride (TMC) (0.5% *w*/*v*, positive control), plotted as a function of time (*n* = 3) (Error bars represent standard deviation (SD)).

**Figure 3:**
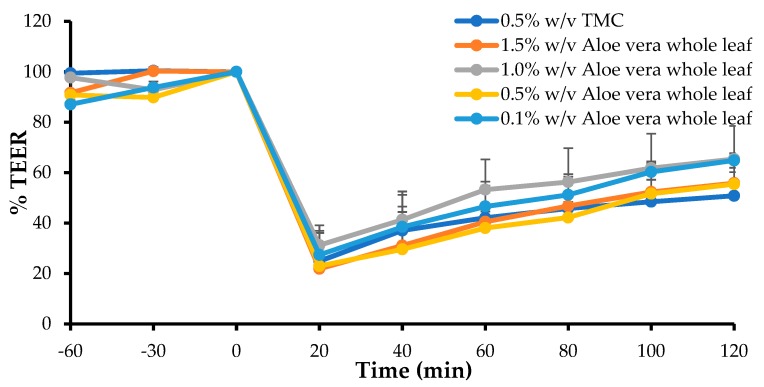
Percentage TEER of the Caco-2 cell monolayers after the application of the *A. vera* whole leaf, at different concentrations, and TMC (0.5% *w*/*v*, positive control) plotted as a function of time (*n* = 3) (Error bars represent SD).

The TEER value of a cell monolayer is indicative of the tight junction integrity and a decrease in TEER has been related to the opening of tight junctions and, therefore, also to an increase in the paracellular permeability [56]. The TEER studies were performed to indicate the capability of the *A. vera* gel and the whole-leaf extract, to open tight junctions. From the results it was clear that maximum TEER reduction was already evident at 20 min, after application of the test solutions, and the TEER started to recover over the 120 min period, towards the initial value. The percentage TEER of the Caco-2 cell monolayers, after application of the test solutions and the positive control (i.e., TMC) plotted as a function of time, are shown in Figure 2, for four different concentrations of the *A. vera* gel, and in Figure 3, for the four different concentrations of the *A. vera* whole-leaf extract.

From Figure 2, it is clear that a rapid (within 20 min) and relatively large decrease in the percentage TEER of Caco-2 cell monolayers occurred after the application of the *A. vera* gel solutions, which was similar in extent to that of the positive control (TMC at 0.5% *w*/*v*), except for the 1.5% *w*/*v A. vera* gel solution, which exhibited a lower TEER reduction effect. The decrease in TEER caused by the *A. vera* gel on the Caco-2 cell monolayers was inversely proportional to the concentration applied. This could probably be explained by the increase in the viscosity of the *A. vera* gel solutions, with each concentration increase, which may have decreased the diffusion of ions across the Caco-2 cell monolayers.

From Figure 3, it is evident that the *A. vera* whole-leaf extract solutions caused a rapid and relatively large decrease in the TEER of the Caco-2 cell monolayers, which started to recover towards the initial value, over the period of 120 min. Furthermore, the decrease in TEER caused by some of the *A. vera* whole-leaf extract solutions, was larger than that of the positive control (0.5% *w*/*v* TMC). However, the reduction in TEER did not correlate, directly or inversely, with the concentration of the *A. vera* whole-leaf extract solutions.

Nonetheless, the TEER reduction results are in line with previous studies on the application of the *A. vera* gel and whole leaf extract on epithelial surfaces [32,33,35]. The TEER results indicated that the *A. vera* gel and whole-leaf extract were capable of opening tight junctions between the Caco-2 cells.

### 3.5. In Vitro Permeation Studies

The *A. vera* gel and whole-leaf extract showed the ability to enhance the transport of the FITC-dextran with a molecular weight of 4 kDa, across the Caco-2 cell monolayers, however, no transport could be detected for the FITC-dextran molecules, with molecular weights of 10 kDa (FD-10), 20 kDa (FD-20), and 40 kDa (FD-40), in the absence or presence of the *A. vera* gel and whole-leaf extract solutions, across the Caco-2 cell monolayers.

**Figure 4:**
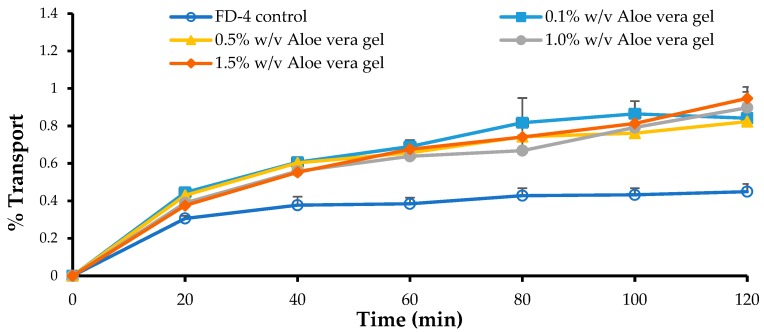
Percentage transport of the Fluorescein isothiocyanate (FITC)-dextran (FD) (FD-4 with MW of 4 kDa) plotted as a function of time, across the Caco-2 cell monolayers, in the absence (FD-4 control) and presence of the *Aloe vera* gel solutions, with different concentrations (*n* = 3; error bars represent SD).

**Figure 5:**
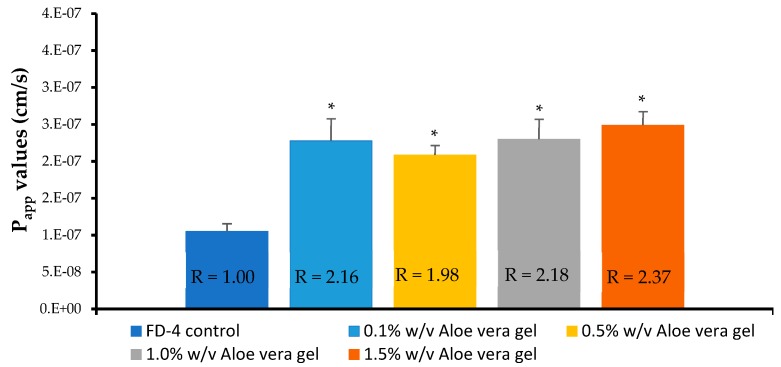
Apparent permeability coefficient (P_app_) values of the FITC-dextran (FD-4 with MW of 4 kDa), across the Caco-2 cell monolayers, when co-applied with the *Aloe vera* gel solutions. Bars marked with an asterisk (*) indicate statistical significant differences from the control (*p* < 0.05) (*n* = 3; error bars represent SD) (R = permeation-enhancement ratio).

**Figure 6:**
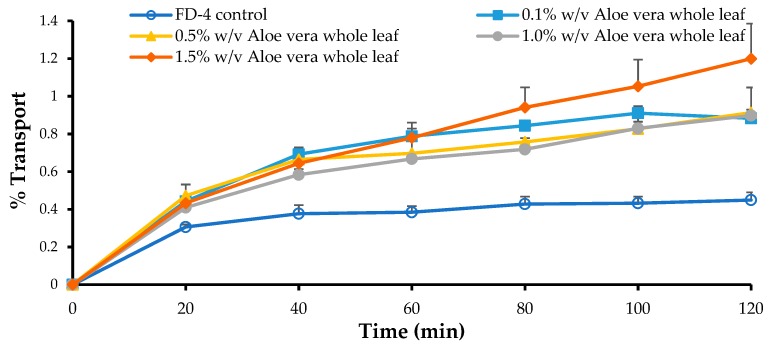
Percentage transport of the FITC-dextran (FD-4 with MW of 4 kDa) plotted as a function of time, across the Caco-2 cell monolayers, in the absence (FD-4 control) and presence of the *Aloe vera* whole-leaf extract solutions, with different concentrations (*n* = 3; error bars represent SD).

**Figure 7:**
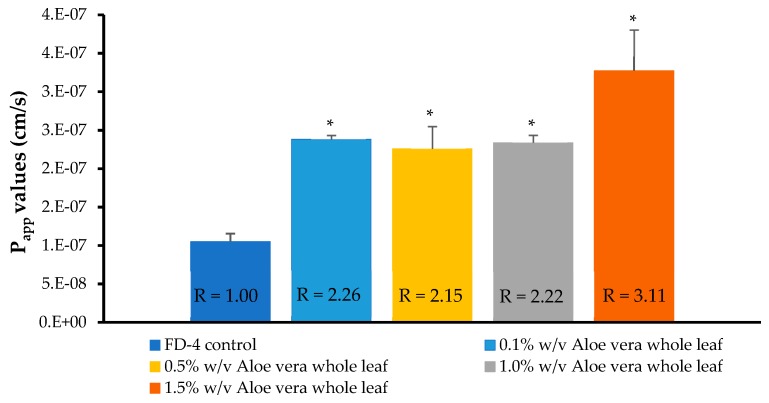
Apparent permeability coefficient (P_app_) values of the FITC-dextran (FD-4 with MW of 4 kDa) across the Caco-2 cell monolayers, when co-applied with the *Aloe vera* whole-leaf extract solutions. Bars marked with an asterisk (*) indicate statistically significant differences from the negative control group (*p* < 0.05) (*n* = 3; error bars represent SD) (R = permeation-enhancement ratio).

The transport curves and apparent permeability coefficient (P_app_) values of the FD-4, across the Caco-2 cell monolayers, in the absence and presence of four different concentrations of the *A. vera* gel and whole-leaf extract, respectively, are shown in Figure 4, Figure 5, Figure 6 and Figure 7.

From Figure 4, a clear increase in the percentage transport of FD-4, in relation to the negative control (FD-4 alone), can be seen for all concentrations of the *A. vera* gel solutions that were applied to the Caco-2 cell monolayers, with the FD-4. The transport of the FD-4, alone, showed an initial increase until 20 min, whereafter it reached a plateau, over the rest of the 120 min transport period. While in the presence of the *A. vera* gel it continued to be transported, albeit at a slower rate than the first 20 min.

A slightly higher than the two-fold increase in the transport of the FD-4 (R or permeation-enhancement ratio values indicated on Figure 5) in relation to the control group (FD-4 alone) was shown for all the concentrations of the *A. vera* gel solutions tested. The transport of the FD-4 was, statistically, significantly higher (*p* < 0.05) in the presence of all the *A. vera* gel solutions, compared to the transport of the control group (FD-4 alone). *A. vera* gel, therefore, showed the ability to significantly enhance the transport of a macromolecule (FD-4), across intestinal epithelial cell monolayers (Caco-2), which is in line with previous findings [32,33,34].

From Figure 6, a distinct increase in the FD-4 transport across the Caco-2 cell monolayers can be seen for all the concentrations of the *A. vera* whole-leaf extract solutions, when compared to the negative control group (FD-4 alone). The transport of the FD-4 alone (negative control) reached a plateau after 20 min, and only slightly increased, further, over the remainder of the transport period (120 min); while in the presence of the *A. vera* whole-leaf extract, it continued to be transported.

The transport of the FD-4 in the presence of all concentrations of the *A. vera* whole-leaf extract solutions, were significantly higher (Figure 7) than that of the control group (FD-4 alone) (*p* < 0.05). *A. vera* whole-leaf extract has, therefore, shown the ability to significantly enhance the transport of a macromolecular model compound (FD-4), across the intestinal epithelial cell monolayers (Caco-2), which is in line with previous findings [32,33,34]. The absorption-enhancing effect of the *A. vera* whole-leaf extract was in agreement with the TEER reduction results and can, therefore, most probably be attributed to its tight junction-modulating activities.

### 3.6. Confocal Laser Scanning Microscopy (CLSM)

#### 3.6.1. Visualization of the Transport Pathway

**Figure 8:**
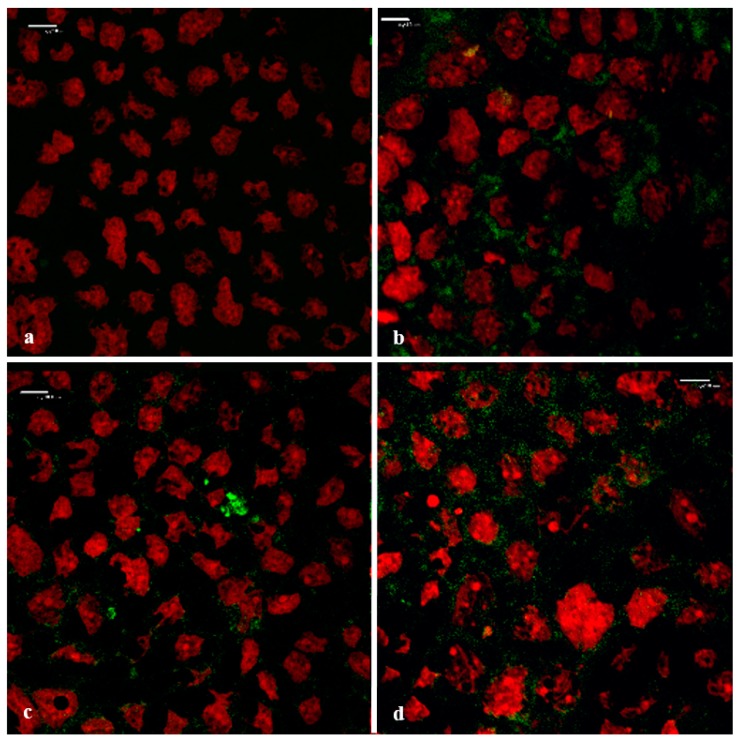
Top-view confocal micrograph images of the Caco-2 cell monolayers on which the FITC-dextran with MW of 4 kDa (FD-4) was applied (**green**: FD-4 and **red**: cell nuclei stained with propidium iodide). (**a**) Negative control (FD-4 alone), (**b**) positive control (0.5% *w*/*v* TMC), (**c**) *A. vera* gel (1.0% *w*/*v*), and (**d**) *A. vera* whole-leaf extract (1.0% *w*/*v*) (Scale bars represents 10 µm).

Figure 8 shows the top-view confocal micrograph images of the Caco-2 cell monolayers to which the FD-4 was applied, in the absence (negative control) and presence of the *A. vera* gel and whole-leaf extract, as well as TMC (positive control).

From the confocal micrograph images in Figure 8, the intercellular accumulation of FD-4 (green) between the Caco-2 cells can be observed, when it was applied with the absorption enhancers (Figure 8b–d), compared to no accumulation in the negative control (Figure 8a). This accumulation in the intercellular spaces between the cells indicated a movement of the FD-4 molecules, via the paracellular pathway. The CLSM image of the positive control (0.5% *w*/*v* TMC, a known tight junction modulator and paracellular absorption enhancer) is in accordance to previously published papers [58,59]. The lack of green fluorescence inside the cells, confirmed that the incubation with TMC, the *A. vera* gel, and *A. vera* whole-leaf extract, did not damage the cell membranes. The paracellular accumulation of the FD-4, in the presence of the *A. vera* gel and the whole-leaf extract, corresponded with the TEER reduction results and is most probably the result of their ability to modulate tight junctions.

#### 3.6.2. Visualization of the F-Actin Filaments in the Cytoskeleton

**Figure 9:**
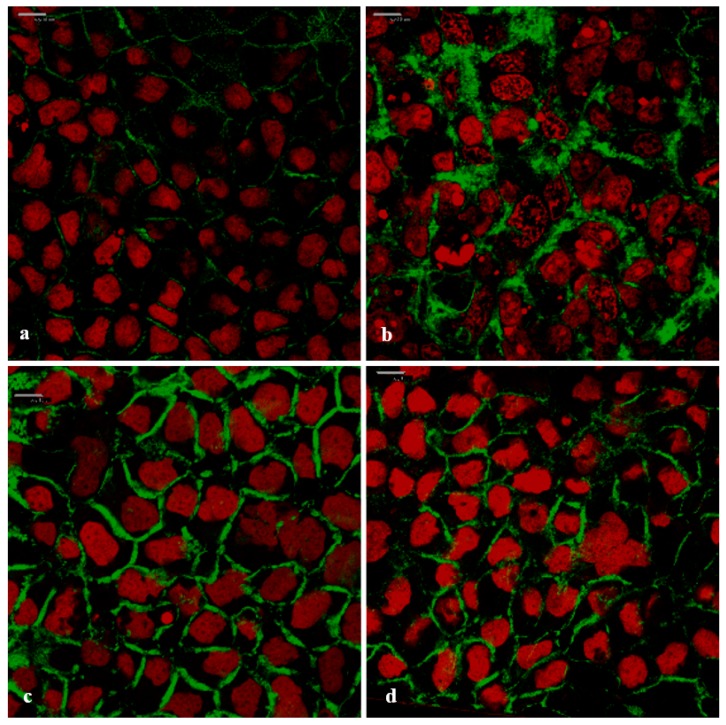
Confocal micrograph images of the filamentous actin (F-actin) distribution in the Caco-2 cell monolayers (**green**: F-actin stained with CytoPainter^®^ Phalloidin iFluor 488 and **red**: cell nuclei stained with propidium iodide). (**a**) Negative control (untreated Caco-2 cell monolayer), (**b**) positive control (0.5% *w*/*v* TMC), (**c**) 1.0% *w*/*v A. vera* gel, and (**d**) 1.0% *w*/*v A. vera* whole-leaf extract (Scale bars represent 10 µm).

According to Ward et al. [11], disruption of the actin cytoskeleton through modulation of the F-actin structure can cause opening of the tight junctions and an increase in paracellular permeability. The re-arrangement of filamentous actin (F-actin) in the cytoskeleton of the Caco-2 cells was visualized, in order to determine the possible mechanism of action by which the *A. vera* gel and the whole-leaf extract increase the paracellular absorption. The CLSM images in Figure 9 show the F-actin expression in a Caco-2 cell monolayer, after incubation with the TMC (positive control), *A. vera* gel, and whole-leaf extract, and without an absorption enhancer (negative control).

In the confocal micrograph images shown in Figure 9, the differences can be seen in the appearance of the F-actin when the Caco-2 cells were treated with *A. vera* gel, *A. vera* whole-leaf extract, and TMC (positive control), as compared to the negative control (untreated cells). The untreated Caco-2 cell monolayer (negative control) showed very little and irregular fluorescence (green) distribution of the F-actin, along the cell borders. In contrast to this, the F-actin fluorescence localization (green) in all the other images was visibly different (Figure 9b–d), which indicates that the F-actin distribution was re-arranged. The rearranged F-actin fluorescence pattern, seen in Figure 9b, is in congruence with previously published research on the effect of TMC (a known tight junction modulator) on the actin cytoskeleton of the Caco-2 cells [56]. The changed fluorescence patterns of the F-actin seen in the Caco-2 cells that were treated with *A. vera* gel (Figure 9c) and the *A. vera* whole-leaf extract (Figure 9d), were similar to that of the TMC. The changed F-actin distribution, therefore, indicates that tight junction modulation occurred in the presence of *A. vera* gel and *A. vera* whole-leaf extract.

## 4. Conclusions

Tight junction modulation by the *A. vera* gel and *A. vera* whole-leaf extract has previously been suggested, by Chen et al. [32], as a possible mechanism of its action for drug-absorption enhancement. The results obtained from this study confirmed tight junction modulation by the *A. vera* gel and *A. vera* whole-leaf extract, by means of different tests, including TEER reduction, transport enhancement of the FD-4, accumulation of FD-4 between the epithelial cells (i.e., in the intercellular spaces), and F-actin disruption, as determined with confocal laser scanning microscopy.

## Figures and Tables

**Table 1:** Required transepithelial electrical resistance (TEER) values, as indicative of the intact Caco-2 cell monolayers, on different Transwell® plate insert membranes.

Type of Transwell^®^ Plate	TEER Value Measured (Ω)	TEER Value Normalized for Surface Area (Ω·cm^2^)
Transwell^®^ 6-well plates (surface area = 4.67 cm^2^) [45]	150	700.5
Transwell^®^ 24-well plates (surface area = 0.33 cm^2^) [46]	750	247.5
Snapwell^®^ 6-well plates (surface area = 1.12 cm^2^) [47]	179	200.0

**Table 2:** Excitation and emission wavelengths of the dyes and transport marker used in the confocal imaging experiments [51,54,57].

Compound	Excitation Wavelength (nm)	Emission Wavelength (nm)
FITC-dextran	494	518
Phalloidin iFluor	493	517
Propidium Iodide	535	615
